# Translation and cross-cultural adaptation of the anchor points of the Cornell Assessment of Pediatric Delirium scale into Portuguese

**DOI:** 10.5935/2965-2774.20230165-en

**Published:** 2023

**Authors:** Marina dos Santos Ramos Barbosa, Lívia Barboza Andrade, Maria do Carmo Menezes Bezerra Duarte, Roberta Esteves Vieira de Castro

**Affiliations:** 1 Instituto de Medicina Integral Professor Fernando Figueira - Recife (PE), Brazil; 2 Rede D’Or São Luiz - Rio de Janeiro (RJ), Brazil

**Keywords:** Delirium, Child, Translating, Intensive care units, pediatric

## Abstract

**Objective:**

To translate and cross-culturally adapt the Cornell Assessment of Pediatric Delirium anchor points from English to Brazilian Portuguese.

**Methods:**

For the translation and cross-cultural adaptation of the anchor points, all steps recommended internationally were followed after authorization for use by the lead author. The stages were as follows: translation of the original version into Portuguese by two bilingual translators who were native speakers of the target language, synthesis of the versions, reverse translation by two translators who were native speakers of the source language, review and synthesis of the back-translation, review by a committee of experts and preparation of the final version.

**Results:**

The translation and cross-cultural adaptation of the anchor points was conducted in accordance with recommendations. The linguistic and semantic issues that arose were discussed by a committee of judges, with 91.8% agreement, as determined using a Likert scale, after changes by consensus. After reanalysis by the authors, there were no changes, resulting in the final version, which was easy to understand and administer.

**Conclusion:**

The translation and cross-cultural adaptation of the anchor points of the Cornell Assessment of Pediatric Delirium scale into Portuguese spoken in Brazil were successful, maintaining the linguistic and semantic properties of the original instrument. The table of anchor points is easy to understand and will be helpful during the assessment of children younger than 24 months using the Cornell Assessment of Pediatric Delirium scale.

## INTRODUCTION

*Delirium* is described as acute brain dysfunction with a fluctuating course and altered consciousness and cognition that may occur in critically ill patients and is associated with an increased duration of mechanical ventilation and length of stay in the intensive care unit (ICU), in addition to a higher risk of mortality.^(1-4)^ Based on clinical presentation, *delirium* can be classified as hyperactive if there is a predominance of agitation; hypoactive, which is characterized by a decreased response to stimuli; and mixed, when there is fluctuation between hypoactivity and hyperactivity symptoms.^(5-7)^

In the pediatric setting, studies suggest that delirium and neurocognitive disorders occur in at least 30% of critically ill children admitted to the pediatric ICU on mechanical ventilator support and report an incidence of 4 to 5%, but it is likely that these numbers are underestimated due to the low sensitivity of the tools used to identify all types of *delirium* in all age ranges.^(5-10)^ These findings were confirmed in a systematic review of the analysis of the prevalence of pediatric *delirium* using validated tools; the conclusion of the review was that pediatric *delirium* occurs in approximately 34% of children admitted to pediatric intensive care units, with the hypoactive subtype being the most prevalent.^(11)^

The most cited risk factors associated with pediatric *delirium* are pain, separation anxiety, absence of a caregiver, admission to the pediatric ICU, mechanical ventilation, anticholinergic medications, sleep deprivation (noise, cold and light), mechanical restraint, number of procedures (placement and removal of devices), and use of sedatives and analgesics.^(7,12,13)^ Among the most cited causes for the development of pediatric *delirium* are cumulative doses of benzodiazepines, opioids, number of sedative classes used, deep sedation and chest surgery.^(7)^

In recent years, several screening tools for use in children in the PICU have been proposed and validated. Among such tools is the Cornell Assessment of Pediatric Delirium (CAPD) scale, a promising clinical triage tool designed and validated for use in the pediatric ICU that is easy to use, allows fast observation, is applicable by a multidisciplinary team and can detect all types of *delirium* in all pediatric age groups.^(13)^

Although it has been translated into Portuguese and culturally adapted for use in the pediatric population of Brazil,^(14)^ the CAPD has not yet been validated for use in the Brazilian population, nor have the anchor points table been translated and cross-culturally adapted, which would aid in the evaluation of children under 2 years of age.^(13)^ Some published studies have reported the translation and cross-cultural adaptation of the CAPD into other languages, including Japanese, Italian and Danish, including validation for use in Denmark.^(15-17)^

The process required for the cross-cultural adaptation of instruments into new languages/cultures involves more than the simple translation of the original and literal comparison with a back-translation. There is no consensus regarding execution strategies, but it is recommended that the process should be meticulous and consider the cultural context and lifestyle of the target population. This process has several advantages compared to the development of a new instrument with the same purpose. In addition to alleviating the lack of available instruments, it can also contribute to the performance of cross-cultural studies, which may provide further clarification and understanding of the subject studied and its specificities in different languages and cultures, allowing the comparison of different populations and the exchange of information, without the bias of cultural and language barriers.^(18-20)^

In this context, the objective of this study was to translate and cross-culturally adapt the table of CAPD anchor points into Portuguese for use in Brazil.

## METHODS

This was a methodological study involving the translation and cross-cultural adaptation of the CAPD anchor points into Brazilian Portuguese. The CAPD is a tool for diagnosing *delirium* in children under intensive care.^(14-17)^ In addition to the scale, the author of the CAPD developed a table with anchor points to aid in the evaluation of *delirium* in children under 24 months of age.^(13)^ The translation and cross-cultural adaptation of the table into Brazilian Portuguese is important and necessary. Although it was translated into Portuguese and culturally adapted for use in the pediatric population of Brazil, the table of anchor points was not translated and cross-culturally adapted (Table 1).^(14)^

**Table 1:** Original version

	Newborn	4 weeks	6 weeks	8 weeks	28 weeks	1 year	2 years
1. Does the child make eye contact with the caregiver?	Fixes on face	Holds gauze briefly Follows 90 degrees	Holds gauze	Follows moving object/ caregiver past midline, regards examiner’s hand holding object, focused attention	Holds gauze. Prefers primary parent. Looks at speaker	Holds gauze. Prefers primary parent. Looks at speaker	Holds gauze. Prefers primary parent. Looks at speaker
2. Are the child's actions purposeful?	Moves head to side, dominated by primitive reflexes	Reaches (with some discoordination)	Reaches	Symmetric movements, will passively grasp handed object	Reaches with coordinated smooth movement	Reaches and manipulates objects, tries to change position, if mobile may try to get up	Reaches and manipulates objects, tries to change position, if mobile may try to get up and walk
3. Is the child aware of his/her surroundings?	Calm awake time	Awake alert time Turns to primary caretaker’s voice May turn to smell of primary care taker	Increasing awake alert time Turns to primary caretaker’s voice May turn to smell of primary care taker	Facial brightening or smile in response to nodding head, frown to bell, coos	Strongly prefers mother, then other family members. Differentiates between novel and familiar objects	Prefers primary parent, then other family members, upset when separated from preferred care takers. Comforted by familiar objects, especially favorite blanket or stuffed animal	Prefers primary parent, then other family members, upset when separated from preferred care takers. Comforted by familiar objects, especially favorite blanket or stuffed animal
4. Does the child communicate needs and wants?	Crisps when hungry or uncomfortable	Crisps when hungry or uncomfortable	Crisps when hungry or uncomfortable	Crisps when hungry or uncomfortable	Vocals/indicates about needs, e.g., hunger, discomfort, curiosity in objects, or surroundings	Use single words or signs	3-4-word sentences, or signs. May indicate toilet needs, calls self or me
5. Is the child restless?	No sustained awake alert state	No sustained calm state	No sustained calm state	No sustained calm state	No sustained calm state	No sustained calm state	No sustained calm state
6. Is the child inconsolable?	Not soothed by parental rocking, singing, feeding, comforting actions	Not soothed by parental rocking, singing, feeding, comforting actions	Not soothed by parental rocking, singing, feeding, comforting actions	Not soothed by parental rocking, singing, comforting actions	Not soothed by usual methods e.g., singing, holding, talking	Not soothed by usual methods e.g., singing, holding, talking, reading	Not soothed by usual methods e.g., singing, holding, talking, reading (May tantrum, but can organize)
7. Is the child underactive - very little movement while awake?	Little if any flexed and then relaxed state with primitive reflexes (child should be sleeping comfortably most of the time)	Little if any reaching, kicking, grasping (still may be somewhat discoordinated)	Little if any reaching, kicking, grasping (may begin to be more coordinated)	Little if any purposive grasping, control of head and arm movements, such as pushing things that are noxious away	Little if any reaching, grasping, moving around in bed, pushing things away	Little if any play, efforts to sit up, pull up, and if mobile crawl or walk around	Little if any more elaborate play, efforts to sit up and move around, and if able to stand, walk, or jump
8. Does it take the child a long time to respond to interactions?	Not making sounds or reflections active as expected (grasp, suck, Moro)	Not making sounds or reflections active as expected (grasp, suck, Moro)	Not kicking or crying with noxious stimuli	Not cooing, smiling, or focusing gauze in response to interactions	Not babbling or smiling/laughing in social interactions (or even actively rejecting an interaction)	Not following simple directions. If verbal, not engaging in simple dialogue with words or jargon	Not following 1-2 step simple commands. If verbal, not engaging in more complex dialogue

The study was initiated after authorization of use by the original author, Dr. Chane Traube, Weil Cornell Medical College, New York, United States, and approval of the study by the Ethics Committee in Research Involving Human Beings of *Instituto de Medicina Integral Professor Fernando Figueira* (IMIP), Recife (PE), under process 5,882.615. The procedures adopted in this study followed the model proposed by Reichenheim & Moraes and involved the following steps: permission from the lead author; translation and concordance; back translation and concordance; analysis by a committee of judges; and review and construction of the final version.^(20)^

### Description of the Cornell Assessment of Pediatric Delirium and table of anchor points

The CAPD is composed of eight items to be observed. Each of the eight items is scored from zero to four, and a total score equal to or greater than nine points indicates the presence of *delirium*. To aid in the evaluation of children younger than 24 months, the author developed a table of anchor points, which are the main developmental milestones, divided into seven columns (newborn, 4 weeks, 6 weeks, 8 weeks, 28 weeks, 1 year and 2 years).^(13)^

### Translation and cross-cultural adaptation

The table of anchor points was translated by experienced bilingual translators. All translators produced independent translations.

The steps for the translation and cultural adaptation process were conducted in accordance with internationally accepted recommendations, namely, authorization from the author of the original version for the translation and cultural adaptation of the anchor points; translation from English into Portuguese by two Brazilian translators fluent in English; synthesis of the translated versions (to evaluate the linguistic, semantic, idiomatic, conceptual and contextual discrepancies, to obtain a single version); back-translation (back-translation of the summary version in Portuguese into the target language - English - by two bilingual translators who are native English speakers and fluent in Portuguese); review and harmonization of the back-translation to produce a single version; meeting with the committee of judges formed by experts with practical experience in the area in question; and after making necessary corrections and adaptations based on feedback from the committee of judges, reconciliation and preparation of the final version.

### Translation into Portuguese and synthesis of the translated versions

The table was translated by two native Portuguese translators with command of English, resulting in two versions. The two versions, which were independently translated, were analyzed and compared during a meeting between the translators and the lead author. A consensus approach was used to resolve any differences, resulting in a single version of the scale in Portuguese.

### Back-translation into English and synthesis of the back-translated versions

The synthesized Portuguese version was translated back into English by two independent translators who were native speakers of English and fluent in Portuguese. The translators were not familiar with the concepts explored in the table, nor were they aware of the original English version. The two versions, which were independently translated, was performed, were synthesized, resulting in a single version of the scale in English.

### Committee of judges

The versions were reviewed and evaluated by a committee of judges composed of 13 professionals who specialized in the content addressed (physical therapists, pediatric intensivists, nurses and neurologists). The purpose of this phase was to resolve disagreements regarding translations: conceptual (referring to the conceptual formulation of the table), idiomatic (different linguistic expressions), semantic (differences related to the content of the table) and experiential (related to cultural differences).^(18-20)^

After the meeting of the committee of judges, a prefinal version of the table of anchor points was produced. Each item was revised, and relevant modifications suggested by the experts were incorporated, producing the final version of the table of anchor points. Thus, the final version of the CAPD anchor points table adapted to Brazilian Portuguese was prepared.

## RESULTS

After the first stage (translation), two Portuguese versions of the table of anchor points were obtained. In the synthesis of the versions, the authors considered a combination of the versions because the translations were similar and different terms were synonyms. In the back-translation of the Portuguese version into English, no changes were made to the words suggested by the translators because there was no discrepancy between the items in the original scale and those in the back-translated version.

The items evaluated and altered, by consensus, by the lead author during the meeting of the expert committee are shown in boldface in table 2. Some words were removed or added to improve agreement and facilitate understanding. Specific terms in the last row of the table underwent the most changes when compared with the terms in the translated version of the original table, but the semantics did not change because the terms (suck/suction), (grasp/grab), and (disturbing/uncomfortable) were considered synonyms. In the elaboration of the final version (Table 3), although the items were considered little changed compared to the items in the original version, these terms were synonymous and, when back-translated into English, were identical to the original (*grasp, suck, noxious stimuli*) and did not change the semantics of the items.

**Table 2:** Version with adjustments after suggestions by the committee of judges

	Newborn	4 weeks	6 weeks	8 weeks	28 weeks	1 year	2 years
1. Does the child make eye contact with the caregiver?	Fixes gaze on the face	Fixes gaze for a short time. Tracks movements up to 90 degrees	Keeps gaze **Fixes gaze**	Follows a moving object/caregiver beyond the midline, considers the examiner’s hand holding the object, focused attention **Maintains attention, follows objects/caregiver moving beyond midline**	Fixes gaze. Prefers primary parent. Looks at who is talking	Fixes gaze. Prefers primary parent. Looks at who is talking	Fixes gaze. Prefers primary parent. Looks at who is talking
2. Are the child’s actions purposeful?	Moves head to the side, dominated by primitive reflexes	Reaches (with some lack of coordination) **Yes, with some incoordination**	Reaches **Yes**	Symmetrical movements, can passively grasp a given object	Yes, with smooth coordinated movements	Reaches and manipulates objects, tries to change position, if mobile, tries to get up **Reaches and manipulates objects. Tries to change position. Moves to try to get up**	Reaches and manipulates objects. Tries to change position. Moves to try to get up and walk **Reaches and manipulates objects, tries to change position, if mobile, tries to get up and walk**
3. Is the child aware of his or her surroundings?	Calm while awake	Alert while awake Turns to the voice of the primary caregiver Can turn around when smelling the main caregiver **Awake and alert. Turns toward the voice of and when smelling the main caregiver**	Increased alertness while awake Turns to the voice of the primary caregiver Can turn around when smelling the main caregiver **Stays alert longer. Turns toward the voice of and when smelling the main caregiver**	Smiling or positive features in response to head nodding, frowning and grimacing	Strongly prefers the mother to other family members. Differentiates new objects from familiar objects	Prefers direct caregivers to others. Gets upset about being separated from the main caregiver. Is comforted by familiar objects such as a favorite blanket or plush	Prefers direct caregivers to others. Gets upset about being separated from the main caregiver. Is comforted by familiar objects such as a favorite blanket or plush
4. Does the child communicate needs and desires?	Cries when hungry or uncomfortable **Cries when hungry or uncomfortable**	Cries when hungry or uncomfortable	Cries when hungry or uncomfortable	Cries when hungry or uncomfortable	Vocalizes/indicates needs, e.g., hunger, discomfort, curiosity	Uses single words or signs	Speaks sentences with 3 to 4 words or signs. May indicate physiological needs, uses “I” or “me”
5. Is the child agitated or restless?	No sustained state of alertness	Does not remain calm on a sustained basis	Does not remain calm on a sustained basis	Does not remain calm on a sustained basis	Does not remain calm on a sustained basis	Does not remain calm on a sustained basis	Does not remain calm on a sustained basis
6. Is the child inconsolable?	Not soothed by the parents by rocking, singing, feeding, comforting actions **Not soothed by parents with comforting actions such as rocking, singing, being fed**	Not soothed by parents with comforting actions such as rocking, singing, being fed **Not soothed by parents with comforting actions such as rocking, singing, being fed**	Not soothed by parents with comforting actions such as rocking, singing, being fed **Not soothed by parents with comforting actions such as rocking, singing, being fed**	Not soothed by parents with comforting actions such as rocking, singing, being fed **Is not soothed by parents with comforting actions such as rocking, singing, being fed**	Not calmed by usual methods, such as singing, holding on the lap or talking	Not calmed by usual methods, such as singing, holding on the lap, talking and reading	Not calmed by usual methods, such as singing, holding on the lap, talking and reading (may throw tantrums, but manages to compose self)
7. Is the child hypoactive? Very little movement during wakefulness?	Little or no flexion, relaxed state with primitive reflexes	Little or no attempt to reach, kick, grab (still somewhat uncoordinated)	Little or no attempt to reach, kick, grab (more coordinated)	Little or no intentional grasping attempts, control of head and arm movements, such as pushing away things that are disturbing **Little or no head and arm control such as pushing things away that are bothersome**	Little or no reaching, grasping, moving around the bed and pushing	Little or no effort to play, sit, stand, crawl or walk	Little or no effort to play, sit, move, stand, walk or jump
8. Does the child take a long time to respond to interactions?	Does not emit sounds or active reflexes as expected (grabbing, sucking, Moro) **Does not emit sounds or active reflexes as expected (grasp, sucking, Moro)**	Does not emit sounds or active reflexes as expected (grabbing, sucking, Moro) **Does not emit sounds or active reflexes as expected (grasp, sucking, Moro)**	Does not kick or cry at disturbing stimuli **Does not kick or cry with uncomfortable stimuli**	Does not respond, does not smile and does not stare in response to interactions	Does not babble or smile/laugh in social interactions (or rejects interactions)	Does not obey simple commands. If verbal, does not initiate simple dialog through words or jargon	Does not obey 2-step commands. If verbal, does not initiate complex dialog

**Table 3:** Final version

	Newborn	4 weeks	6 weeks	8 weeks	28 weeks	1 year	2 years
1. Does the child make eye contact with the caregiver?	Fixes gaze on the face	Fixes gaze for a short time. Tracks movements up to 90 degrees	Fixes the gaze	Maintains attention, follows objects/caregiver moving beyond midline	Fixes gaze. Prefers primary parents. Looks at who is talking	Fixes gaze. Prefers primary parent. Looks at who is talking	Fixes gaze. Prefers primary parent prefer. Looks at who is talking
2. Are the child’s actions purposeful?	Moves head to the side, dominated by primitive reflexes	Yes, with some incoordination	Yes	Symmetrical movements, passively grasps a given object	Yes, with smooth coordinated movements	Reaches and manipulates objects. Tries to change position. Moves to try to get up	Reaches and manipulates objects, tries to change position, if mobile, tries to get up and walk.
3. Is the child aware of his or her surroundings?	Calm while awake	Awake and alert. Turns toward the voice of and when smelling the main caregiver	Stays alert longer. Turns toward the voice of and when smelling the main caregiver	Smile or positive features in response to head nodding, frowning and grimacing	Strongly prefers the mother to other family members. Differentiates new objects from familiar objects	Prefers direct caregivers to others. Gets upset about being separated from the main caregiver. Is comforted by familiar objects such as a favorite blanket or plush	Prefers direct caregivers to others. Gets upset about being separated from the main caregiver. Is comforted by familiar objects such as a favorite blanket or plush
4. Does the child communicate needs and desires?	Cries when hungry or uncomfortable	Cries when hungry or uncomfortable	Cries when hungry or uncomfortable	Cries when hungry or uncomfortable	Vocalizes/indicates needs, e.g., hunger, discomfort, curiosity	Uses single words or signs	Speaks sentences with 3 to 4 words or signs. May indicate physiological needs, uses “I” or “me”
5. Is the child agitated or restless?	No sustained state of alertness	Does not remain calm on a sustained basis	Does not remain calm on a sustained basis	Does not remain calm in a sustained manner	Does not remain calm on a sustained basis	Does not remain calm on a sustained basis	Does not remain calm on a sustained basis
6. Is the child inconsolable?	Not soothed by parents with comforting actions such as rocking, singing, being fed	Not soothed by parents with comforting actions such as rocking, singing, being fed	Not soothed by parents with comforting actions such as rocking, singing, being fed	Not soothed by parents with comforting actions such as rocking, singing, being fed	Not calmed by usual methods, such as singing, holding on the lap or talking	Not calmed by usual methods, such as singing, holding on the lap, talking and reading	Not calmed by usual methods, such as singing, holding on the lap, talking and reading (may throw tantrums, but manages to compose self)
7. Is the child hypoactive? Very little movement during wakefulness?	Little or no flexion, relaxed state with primitive reflexes	Little or no attempt to reach, kick, grab (still somewhat uncoordinated)	Little or no attempt to reach, kick, grab (more coordinated)	Little or no head and arm control such as pushing things away that are bothersome	Little or no reaching, grasping, moving around the bed and pushing	Little or no effort to play, sit, stand, crawl or walk	Little or no effort to play, sit, move, stand, walk or jump
8. Does the child take a long time to respond to interactions?	Does not emit sounds or active reflexes as expected (grasp, sucking, Moro)	Does not emit sounds or active reflexes as expected (grasp, sucking, Moro)	Does not kick or cry with uncomfortable stimuli	Does not respond, does not smile and does not stare in response to interactions	Does not babble or smile/laugh in social interactions (or rejects interactions)	Does not obey simple commands. If verbal, does not initiate simple dialog through words or jargon	Does not obey 2-step commands. If verbal, does not initiate complex dialog

During the expert committee meeting, the participants were invited to fill out a form to assess the degree of agreement. After analyzing the results, agreement was 91.8% (Likert scale).

## DISCUSSION

Herein, the process of the translation and cross-cultural adaptation of the CAPD anchor points table from English to Brazilian Portuguese is described. The steps were performed in accordance with the recommendations found in the literature. The linguistic and semantic equivalences between the original table and the Brazilian Portuguese version were satisfactory, as there was no divergence.

The translation and cross-cultural adaptation process is meticulous and necessary, and characteristics of the original version must be preserved. Such adaptation is important due to the heterogeneity of the population and the use of several regional terms. ^(20)^ The Portuguese version of the CAPD anchor points table produced in this study is technically and semantically equivalent to the original version. The evaluation of equivalence between the items in the original scale, the syntheses of the versions translated into Portuguese and the synthesis of the back-translations allow confirmation that most of the items, both in the translation and in the back-translation, are similar to the original version, with small changes. The fact that there were no completely altered terms in the analysis of item equivalences is due, in our opinion, to the simplicity of the instrument, which contains practical terms and uses simple language.

During the evaluation by the committee of multidisciplinary experts, there were some questions about agreement and some specific terms, which were changed by consensus, maintaining the semantic characteristics of the original version. The main changes were made in the last row of the first column (NB) and in the second column (4 weeks), and the term “suck” was replaced with “suction”, and “grab” was replaced with “grasp” because they are usually considered more common terms used in daily practice. In the other columns, some words were modified to improve agreement and understanding; for example, “perturbam” (“perturb”) was replaced with “incomodam” (“annoy”); “disturbing” was replaced with “uncomfortable”; “mantém” was replaced with “fixed”; “lack of coordination” was replaced with “incoordination”; and “moves” was replaced with “mobile”. The changes were made by consensus, taking into account the opinion of the experts and the goal of maintaining the semantic characteristics of the original instrument.

Although there is no gold standard to be strictly followed in the transcultural translation and adaptation process, some guidelines are recommended as essential steps to be followed for such studies.^(20)^ After the small adjustments suggested by consensus during the meeting of the committee of experts and the final meeting with the translators and authors, the final version of the table of CAPD anchor points for Brazilian Portuguese was created.

## CONCLUSION

The table of anchor points within the Cornell Assessment of Pediatric Delirium was translated and cross-culturally adapted to Brazilian Portuguese and is ready to be tested on a larger scale. To this end, further studies are needed to evaluate the psychometric properties of the Cornell Assessment of Pediatric Delirium, making its use possible in all regions of Brazil.

## References

[1] Dervan LA, Di Gennaro JL, Farris RW, Watson RS. (2020). Delirium in a tertiary PICU: risk factors and
outcomes. Pediatr Crit Care Med.

[2] Traube C, Silver G, Reeder RW, Doyle H, Hegel E, Wolfe HA (2017). Delirium in critically ill children: an international point
prevalence study. Crit Care Med.

[3] Castro RE, Rodíguez-Rubio M, Magalhães-Barbosa MC, Prata-Barbosa A. (2021). Delirium pediátrico em tempos da COVID-19. Rev Bras Ter Intensiva.

[4] Silver G, Traube C, Gerber LM, Sun X, Kearney J, Patel A (2015). Pediatric delirium and associated risk factors: a single-center
prospective observational study. Pediatr Crit Care Med.

[5] European Delirium Association; American Delirium Society (2014). The DSM-5 criteria, level of arousal and delirium diagnosis:
inclusiveness is safer. BMC Med.

[6] Dechnik A, Traube C. (2020). Delirium in hospitalised children. Lancet Child Adolesc Health.

[7] Faria RS, Moreno RP. (2013). Delirium na unidade de cuidados intensivos: uma realidade
subdiagnosticada. Rev Bras Ter Intensiva.

[8] Lago PM, Molon ME, Piva JP., Associação de Medicina Intensiva Brasileira (2009). Delirium e agitação psicomotora em UTI
pediátrica. Programa de Atualização em Terapia Intensiva
Pediátrica - PROTIPED.

[9] Creten C, Van Der Zwaan S, Blankespoor RJ, Leroy PL, Schieveld JN. (2011). Pediatric delirium in the pediatric intensive care unit: a
systematic review and an update on key issues and research
questions. Minerva Anestesiol.

[10] Harris J, Ramelet AS, van Dijk M, Pokorna P, Wielenga J, Tume L (2016). Clinical recommendations for pain, sedation, withdrawal and
delirium assessment in critically ill infants and children: an ESPNIC
position statement for healthcare professionals. Intensive Care Med.

[11] Semple D, Howlett M, Strawbridge J, Breatnach CV, Hayden JC. (2022). A systematic review and pooled prevalence of delirium in
critically ill children. Crit Care Med.

[12] Hughes CG, Pandharipande PP, Ely EW (2020). Nashville.

[13] Traube C, Silver G, Kearney J, Patel A, Atkinson TM, Yoon MJ (2014). Cornell Assessment of Pediatric Delirium: a valid, rapid,
observational tool for screening delirium in the PICU. Crit Care Med.

[14] Barbosa MS, Duarte MC, Bastos VC, Andrade LB. (2018). Tradução e adaptação transcultural da
escala Cornell Assessment of Pediatric Delirium para língua
portuguesa. Rev Bras Ter Intensiva.

[15] Hoshino H, Matsuishi Y, Shimojo N, Enomoto Y, Kido T, Inoue Y. (2017). Development of the Japanese version of the Cornell Assessment of
Pediatric Delirium. Acute Med Surg.

[16] Simonsen BY, Lisby M, Traube C, Skovby P. (2019). The Cornell Assessment of Pediatric Delirium: translation and
inter-rater reliability in a Danish pediatric intensive care
unit. Acta Anaesthesiol Scand.

[17] Simeone S, Rea T, Gargiulo G, Esposito MR, Guillari A, Traube C (2019). Cornell Assessment of Pediatric Delirium: Italian cultural
validation and preliminary testing. Prof Inferm.

[18] Costa RM, Cardinot TM, Oliveira LP. (2020). Etapas para validação de instrumentos de
avaliação da qualidade de vida. Revista Científica Multidisciplinar Núcleo do
Conhecimento.

[19] Silva NR, Felipini LM. (2018). Tradução e adaptação transcultural de
instrumentos de avaliação em Fonoaudiologia para o
português brasileiro: uma análise das
diretrizes. TradTerm.

[20] Reichenheim ME, Moraes CL. (2007). Operationalizing the cross-cultural adaptation of epidemological
measurement instruments. Rev Saúde Pública.

